# Structural Elucidation of a Novel Polysaccharide from *Pseudostellaria heterophylla* and Stimulating Glucose Uptake in Cells and Distributing in Rats by Oral

**DOI:** 10.3390/molecules21091233

**Published:** 2016-09-14

**Authors:** Jinlong Chen, Wensheng Pang, Wentao Shi, Bin Yang, Yongjun Kan, Zhaodong He, Juan Hu

**Affiliations:** 1 The Institute of Drug Research, Fujian Academy of Traditional Chinese Medicine, Fuzhou 350003, China; hzf_ketizu@163.com (J.C.); cld_ketizu@163.com (Y.K.); hzd_fjtcmyjy@163.com (Z.H.); 2 The College of Pharmacy, Fujian University of Traditional Chinese Medicine, Fuzhou 350122, China; pws@fjtcm.edu.cn (W.P.); 13860662716@163.com (W.S.); 13905915817@163.com (B.Y.)

**Keywords:** *Pseudostellaria heterophylla*, a novel polysaccharide, structure, glucose uptake, distribution taken orally

## Abstract

The semi-refined polysaccharide of *Pseudostellaria heterophylla* is a complex polysaccharide that exhibits significantly hypoglycemic activities. A novel homogeneous polysaccharide, named as H-1-2, was isolated from the semi-refined polysaccharide. The mean molecular weight of H-1-2 was 1.4 × 10^4^ Da and it was only composed of d-glucose monosaccharide. Structure elucidation indicated that H-1-2 contains pyranride, and has the characteristics of the α-iso-head configuration, a non-reducing end (T-), 4-, 1,6-, and 1,4,6-connection, in all four ways to connect glucose. H-1-2 was a type of glucan, where chemical combination exists in the main chain between 1→4 linked glucose, and contains a small amount of 1,6-linked glucose, which was in the branched chain. In vitro HepG2, 3T3-L1, and L6 cells were used to assess cellular glucose consumption and cellular glucose uptake by glucose oxidase, and the transport of 2-NBDG fluorescence probe results showed that H-1-2 could clearly increase glucose uptake and utilization in muscle and adipose cells, which is beneficial to screen for in the discovery of anti-diabetes lead compounds. H-1-2 was labeled with radioisotopes (^99m^Tc-pertechnetate). ^99m^Tc-labeled-H-1-2 was performed by SPECT/CT analysis images after oral administration in rats. At 4 h post ingestion, about 50% of the radioactivity was observed in the intestine. No significant radioactivity was found in the heart, liver, and kidney, conjecturing that absorption of ^99m^Tc-labeled H-1-2 might, via intestinal mucosa, be absorbed into systemic circulation. This problem, as to whether the polysaccharide is absorbed orally, will need further examination.

## 1. Introduction

*Pseudostellaria heterophylla* (Miq.) Pax ex Pax et Hoffm. is known commonly as Tai Zi Shen (TZS); however, it is also known as Hai Er Shen. TZS, as described in the Pharmacopoeia of People’s Republic of China, is a mild medicine that is used in Chinese medicine to tone the body; it tonifies the qi and generates yin fluids [1]. TZS is restorative in lung damage due to excess heat or dryness, including hot or dry asthma, bronchitis, dry cough, and emphysema [2]. *Pseudostellaria heterophylla* is used to treat the deficient constitution associated with diabetes mellitus, and is usually a core drug for herbal remedies [3,4].

The constituents of TZS are amino acids, fatty acids, heterophyllin, trace elements, polysaccharides, etc. The polysaccharide fractions of TZS have in vitro anti-tumor properties [5]; crude polysaccharide can improve immunity [6]. The polysaccharides of TZS have an excellent effect on hypoglycemic mice and rat, and could significantly improve the general status of diabetic rats, prevent reduction of body weight, and can lower the levels of blood glucose and blood lipids (total cholesterol and triglycerides) [7,8]. TZS polysaccharide could potentially improve the pathology of pancreatic damage in diabetic mice [9], significantly decrease levels of blood glucose, and improve glucose tolerance and blood insulin levels [10]. However, the above-mentioned research activities were restricted to crude polysaccharide of TZS.

Recent work from the authors’ research group found that crude polysaccharide of TZS were degraded to get three, low-molecular-weight fractions, which were then given to type 1 diabetic mellitus (T1DM) mice and type 2 diabetic mellitus (T2DM) obese rats, and the results suggest that the hypoglycemic effects related to molecular size of the polysaccharide were more effective against T2DM than T1DM. T2DM rats receiving daily oral doses of polysaccharides (100~400 mg/kg), 50~210 kDa in molecular weight (named PF40), could, not only significantly lower blood sugar, but also reduce total triglyceride levels in serum, and improve insulin tolerance, regulated by the expression of some biomarkers, including TNF-α (Tumor necrosis factor-α), IL-10 (Interleukin-10), Acrp30 (Adipocyte complement-related protein of 30 kDa), etc. [11]. PF40 was the active anti-T2DM fraction of the polysaccharide. Therefore, it is meaningful to investigate the relationships between chemical structure and the above-mentioned hypoglycemic activities.

Glucose is the most important metabolic fuel, and glucose homeostasis is maintained predominantly by insulin by balancing peripheral glucose uptake and hepatic glucose production. However, if whole body glucose homeostasis is disrupted, hyperglycemia and various physiopathologies associated with metabolic disturbances may occur. The removal of excess glucose from the circulation involves the stimulation of glucose transport into metabolic tissues, and it has become clear that glucose intolerance in type 2 diabetes is manifested by defects in glucose transport into these metabolic tissues. Moreover, adipose tissue was believed to play key roles in glucose homeostasis. In addition to insulin, various hormones and physiological conditions are capable of stimulating glucose uptake. For example, the activation of α_1_-adrenergic or endothelin A receptors results in enhanced glucose uptake rates independently of insulin levels, and physical exercise also plays an important role in the metabolism of glucose by skeletal muscle. Hence, exercise or drug-mediated glucose uptake, are therapeutic targets in type 2 diabetes [12,13].

In this paper, a novel polysaccharide, namely H-1-2, was purified and isolated from PF40. H-1-2 was identified through gel performance chromatography (GPC), determination of the composition of the monosaccharide; determination of the sequence of the polysaccharide using periodate oxidation, smith hydrolysis, infrared spectroscopy, gas-chromatography-mass spectrum (GC-MS), and nuclear magnetic resonance spectroscopy (NMR) experiments. The results provided the first report on the structure of polysaccharide H-1-2, which can be further used in the investigation of cellular glucose consumption; additionally, promoting glucose uptake into cell is beneficial to screen or discovery anti-diabetes lead compounds. To track the pathway of polysaccharide absorption into systemic circulation, the distribution of H-1-2 was investigated using SPECT/CT (single-photon emission computed tomography) analysis images, after oral administration in rats.

## 2. Results and Discussion

### 2.1. Isolation and Purification of Pseudostellaria heterophylla Polysaccharides

PF40, an anti-T2DM active fraction of polysaccharide from TZS was extracted according to literature by the authors’ research group [11]. Fraction H-1 (757 mg) and H-2 (130 mg) were eluted with water from PF40 (7 g) using first-step DEAE-cellulose 52 column chromatography; a novel polysaccharide, designated as H-1-2, was eluted with water from H-1 using a second-step Sephacryl S-300 propylene dextran gel column. The weight of this H-1-2 component was collected and calculated to be 168 mg and was considered a main component of the elution.

After dialysis and lyophilization, H-1-2 showed no absorption at 280 or 260 nm in the UV spectrum. Review of purity results showed that the polysaccharide contained low levels of proteins and nucleic acids. Purity was >97.2%; hence, it was a pure polysaccharide substance and used for further analyses.

### 2.2. Molecular Weight, Specific Optical Rotation and Monosaccharide Composition Analysis

**Figure 1:**
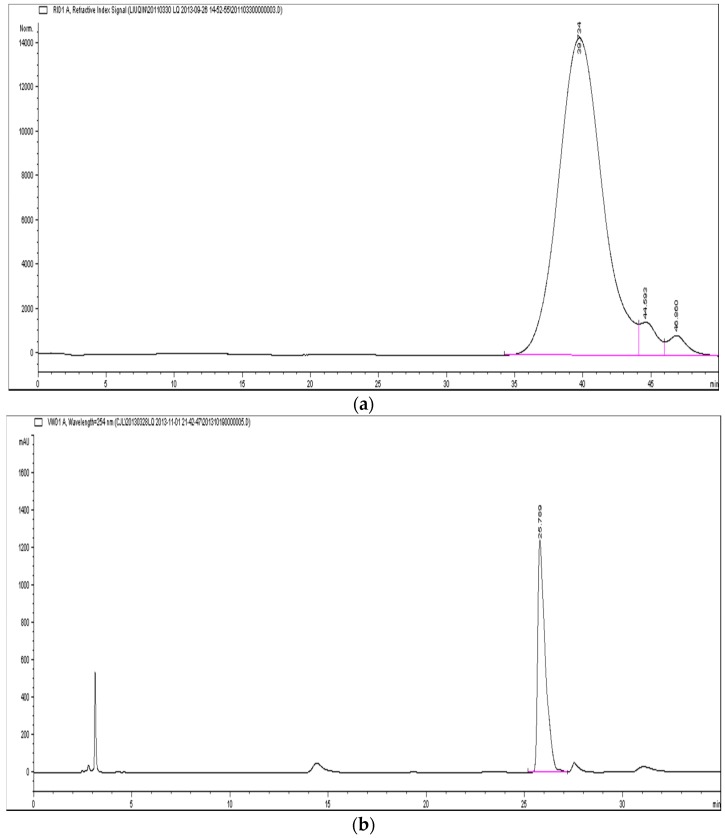
HPGPC (high gel performance chromatography) profile and HPLC (high performance liquid chromatography) profile of polysaccharide H-1-2: (**a**) the chromatogram of H-1-2 by HPGPC analysis; and (**b**) the chromatogram of monosaccharide composition of H-1-2 by HPLC analysis.

H-1-2 had an estimated molecular weight of 1.4 × 10^4^ Da, according to high-performance gel permeation chromatography (HPGPC) results (Figure 1a), and had a specific rotation of [α]${}_{D}^{25}$ + 153.0° (*c* = 0.510, H_2_O). Monosaccharide composition analysis by the high performance liquid chromatography (HPLC) pre-column derivatization method, using 1-phenyl-3-methyl-5-pyrazolone (PMP), showed that H-1-2 contained only d-glucose (Figure 1b); thus, H-1-2 was a polydextrose. Adding Iodine-KI reagent to the solution of H-1-2 showed that a blue–black color resulted and the chemical structure of the H-1-2 polysaccharide resembled that of amylase starch. This is consistent with a larger and positive specific optical rotation.

### 2.3. FT-IR Spectra Analysis

**Figure 2:**
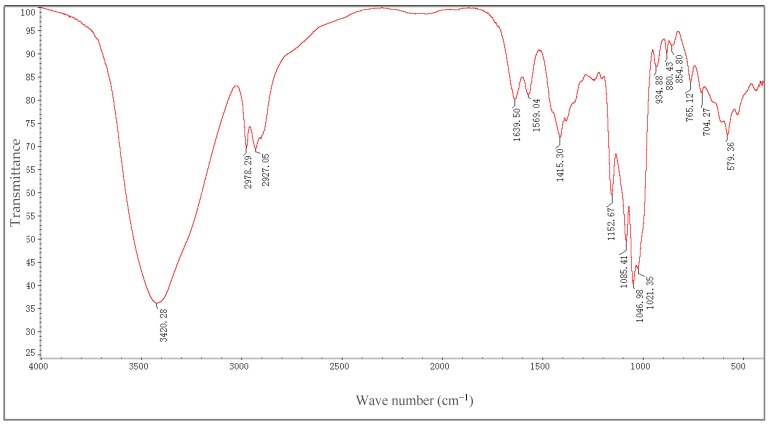
The infrared spectra of H-1-2.

The FT-IR (Fourier Transform Infrared spectrum) of H-1-2 (Figure 2) displayed a strong absorption peak at 3420.28 cm^−1^, which was due to the hydroxyl stretching vibration of the polysaccharide; the peak at 2927.05 cm^−1^ was due to the C–H stretching vibration band. The absorption peak was at 1639.50 cm^−1^, which is a characteristic of hydrated hydroxyls. The absorption peak at 1415.30 cm^−1^ is a characteristic of exocylic C–O, but the peak at 1021.35 cm^−1^ was due to the C–O ring stretching vibration. The FT-IR spectrum displayed a weak absorption peak at 854.80 cm^−1^. The structure of H-1-2 contains pyranride and has the characteristics of an α-iso-head configuration. Near 1730 cm^−1^ showed no absorption of glucuronic acid, suggesting that H-1-2 may be a neutral polysaccharide.

### 2.4. Methylation Analysis

Methylation analysis of polysaccharides was used to investigate the inter-glycosidic linkages between monosaccharide residues. The FT-IR analysis of the polysaccharide methylation shows a hydroxyl peak at 3420.28 cm^−1^, which disappears, indicated that the methylation reaction has been completed. The fully-mentholated products were hydrolyzed, converted into partially methylated alditol acetate (PMAAs), and analyzed using GC-MS (see Table 1). The fragmentation patterns show that H-1-2 contains non-reducing ends and (T-), 1,4-, 1,6-, and 1,4,6-connections, four ways to connect glucose. T- and 1,4,6-connections indicated the presence of branching in the sugar chain. The 1,4-connection glucose residue ratio reached 78.9%, which was primarily the linkage mode. The 1,4,6-connection amounts to 6.7% and demonstrated that the polysaccharide was a main chain 6-substituted glucose residues.

### 2.5. Nuclear Magnetic Resonance Spectroscopy Analysis

**Figure 3:**
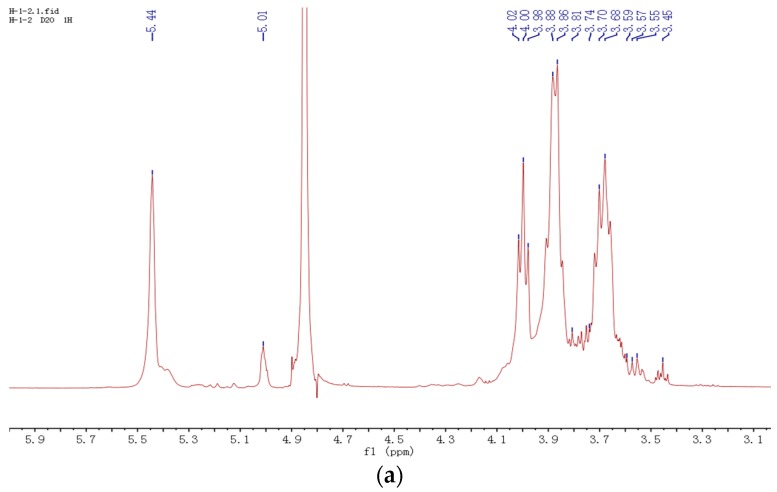

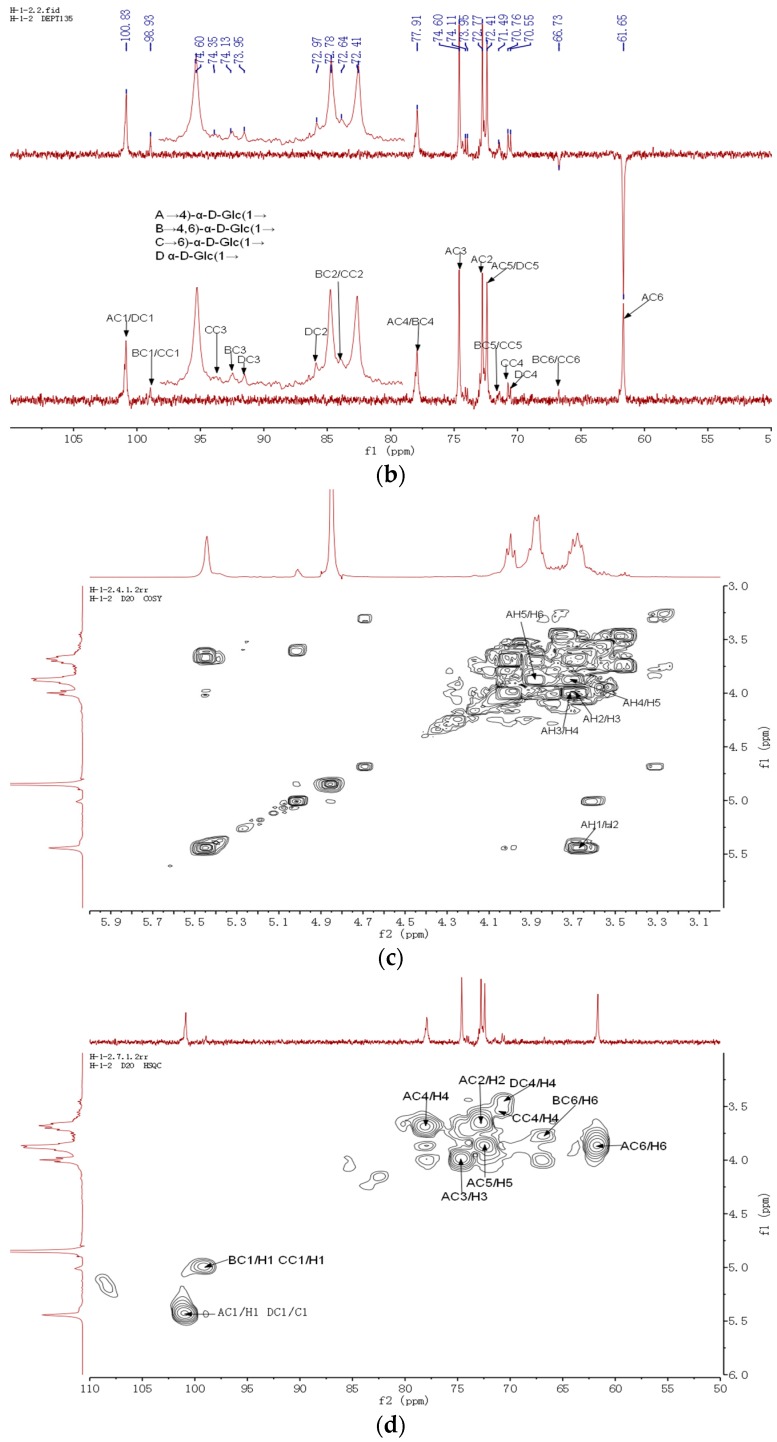

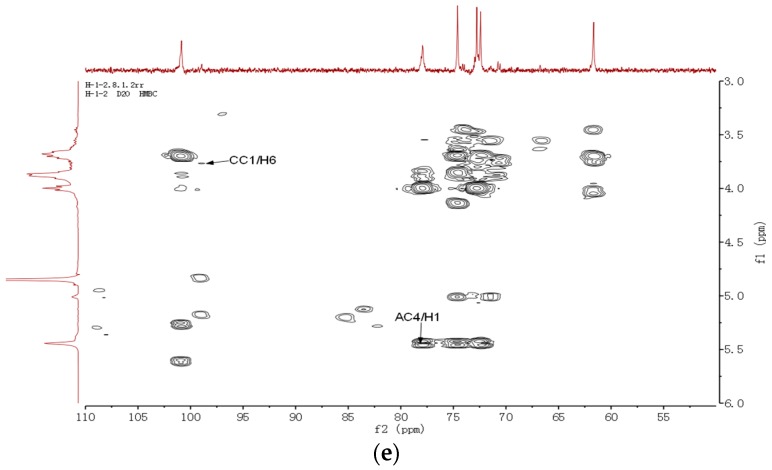
The NMR spectroscopy of H-1-2: (**a**) ^1^H-NMR spectroscopy of H-1-2; (**b**) ^13^C-NMR spectroscopy of H-1-2; (**c**) H-HCOSY spectroscopy of H-1-2; (**d**) HSQC spectroscopy of H-1-2; and (**e**) HMBC spectroscopy of H-1-2.

The ^1^H-NMR (nuclear magnetic resonance hydrogen spectrum) (500 MHz) of H-1-2 (Figure 3a) showed two signals, anomeric protons at δ 5.44 ppm and δ 5.01 ppm. Referring to previously-reported work, the chemical shift value of the anomeric hydrogen of alpha pyranose glucose residues was larger than 5 ppm; conversely, it was less than 5 ppm in beta pyranose glucose residues [14,15,16,17]. The peak of δ 5.44 could be assigned to the anomeric protons of terminal and 1,4-linked glucosidic residues, and the signal of δ 5.01 corresponded to the protons of the 1,6-linked and 1,4,6-linked glucosidic residues.

The ^13^C-NMR spectrum (125 MHz) of H-1-2 (Figure 3b) results showed that two signals of anomeric carbons were also observed at δ 100.83 ppm and δ 98.93 ppm. All anomeric carbon signals were lower than δ 103 ppm, indicating that the polysaccharide was a α-d-glucan, which further validated the results of the IR and ^1^H-NMR spectrums. The lower field signal (δ 100.83) was assigned to the C-1 of the terminal and 1,4-linked glucosidic residues, and the higher field signal (δ 98.93) was the C-1 of 1,6- and 1,4,6-linked glucosidic residues [18,19]. The ^13^C-NMR spectrum showed two methane signals at δ 66.73 ppm and δ 61.65 ppm, which suggested the presence of 6-α-substituted glucosidic residues. The peak of δ 61.65 could be assigned to the C-6 of the terminal and 1,4-linked glucosidic residues, and the signal of δ 66.73 corresponded to the C-6 of the 1,6-linked and 1,4,6-linked glucosidic residues. These results are in agreement with methylation analyses.

2D-NMR spectrums involving ^1^H-^1^H-COSY (homonuclear chemical shift correlation spectroscopy, Figure 3c), ^1^H-^13^C HSQC (heteronuclear single-quantum correlation, Figure 3d), and HMBC (heteronuclear multiple-bond correlation, Figure 3e) were used to identify the chemical shifts and anomeric configurations of the sugar residues present in the repeating units. The results revealed the cross peaks between the anomeric proton of δ 5.44 ppm and the anomeric carbon of δ 100.83 ppm and between the anomeric proton of δ 5.01 ppm and the anomeric carbon of δ 98.93 ppm. The down-field chemical shift of the C-1 at the δ 100.83 carbon signal indicated that the residue was a (1→4)-α-d-glucose. Therefore, the chemical shift of the δ 98.93 carbon signal came from the C-1 of 1,6- and 1,4,6-linked glucose. Cross peaks between δ 3.70 and 72.77, δ 4.0 and 74.60, δ 3.68 and 77.91, and δ 3.86 and 72.41 were observed from the H-HCOSY and HSQC spectrums; these chemical shift values correspond to H-2/C-2, H-3/C-3, H-4/C-4, and H-5/C-5 of 1→4 linked glucose, respectively. The sequence of glycosyl residues in H-1-2 was determined from the results of HMBC and the spectrum revealed cross peaks between the H-6 of 1,4,6-connection and the C-1 of 1,6-linked glucose; as a result, a small amount of 1,6-linked glucose was in the branched chain. The ^1^H-NMR and ^13^C-NMR chemical shift correlation values of H-1-2 are shown in Table 2.

Elemental analysis indicates that the polysaccharide H-1-2 mainly consists of three kinds of elements, Carbon, hydrogen, and oxygen; contains 39.10% carbon and 6.77% hydrogen; and does not contain nitrogen or sulfur. C:H:O = 1:2.08:1.04 is in accordance with the chemical formula of polysaccharides.

**Figure 4:**
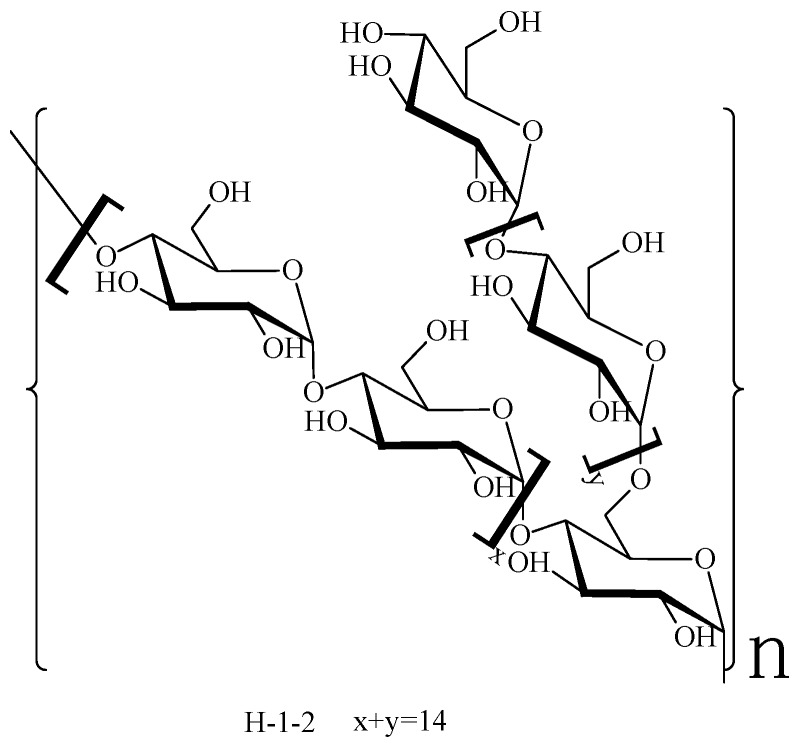
The possible structure unit of polysaccharide H-1-2.

Polysaccharide H-1-2 is a type of glucan where a chemical combination exists in the main chain between 1→4 linked glucose, and contains a small amount of branches. The structure of polysaccharide H-1-2 is inferred and shown in Figure 4.

### 2.6. Assessment of the Anti-Diabetic Activities of Polysaccharide H-1-2 Using Cultured Cell Models

#### 2.6.1. Effects of H-1-2 on Cellular Glucose Consumption

**Figure 5:**
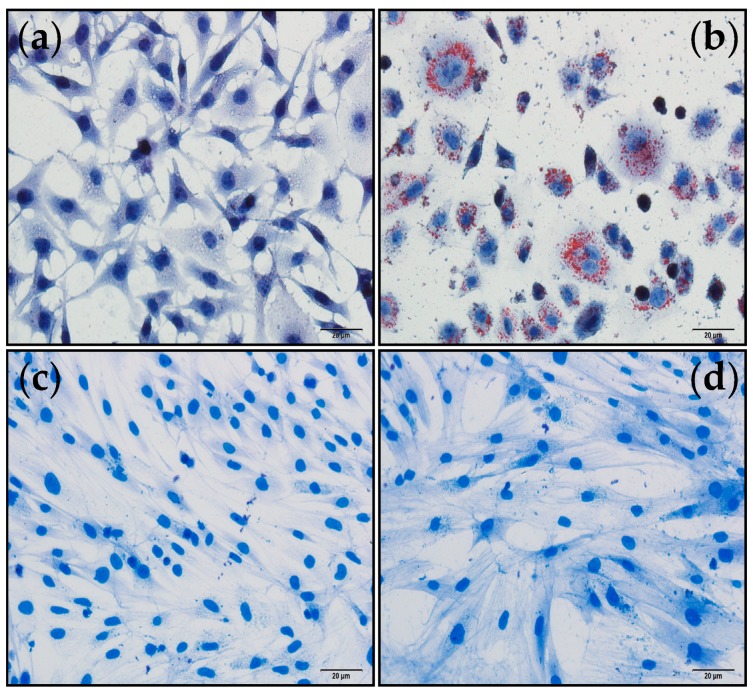
Before and after differentiation of 3T3-L1 and L6 cell lines (Scale bar = 20 μm): (**a**) before differentiation of 3T3-L1 adipocytes strained with Oil Red O; (**b**) after differentiation of 3T3-L1 adipocytes strained with Oil Red O, the cells under microscope were found to exhibit the phenotype of mature adipocytes with many “ring-like” lipid droplets; (**c**) before differentiation of L6 cell line, strained with Coomassie Brilliant Blue; and (**d**) after differentiation of L6 cell line strained with Coomassie Brilliant Blue, to exhibit myotube-like structure.

To investigate the effects of polysaccharide H-1-2 on glucose consumption, three cultured cells models were used: HepG2 (human liver hepatocellular), 3T3-L1 (mouse pre-adipose embryo fibroblast), and L6 (rat skeletal muscle cell line). Proliferation of the three cell types was detected using 3-(4.5-dimethyl-2-thiazoly1)-2,5-diphenyl-2H-terazolium bromide (MTT) assay. On Days 7–15, 3T3-L1 pre-adipocytes were induced to differentiate into fat cells using 10 μM of dexamethasone, 0.5 μM of 3-isobuty-1-methy-xanthine, and 1.0 μg/mL of insulin together. 3T3-L1 cells were stained with Oil Red O to assess the accumulation of lipid droplets, and spectrophotometry was used to measure the lipid content. More than 90% of the cells under microscope were found to exhibit the phenotype of mature adipocytes, with many “ring-like” lipid droplets on Day 10 of differentiation (Figure 5a,b). The L6 cell line was cultured with DMEM containing 2% FBS medium for 14 days, until the appearance of myotube-like structures could be observed using Coomassie Brilliant Blue staining. The myotube-like structures could only be found after L6 cell line differentiation (Figure 5c,d).

**Figure 6:**
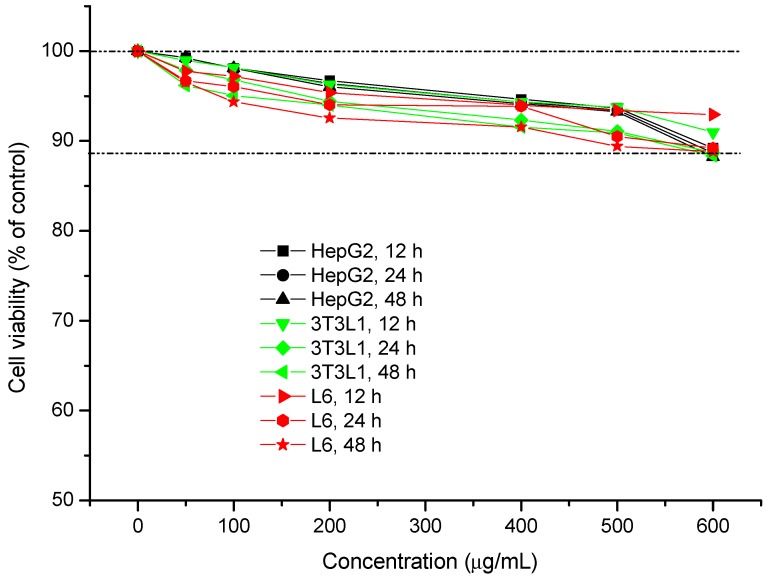
The influences of polysaccharide H-1-2 on the cellular viability of HepG2, 3T3L1, and L6 cell lines, at different concentrations and different times.

Cellular viability was determined using the MTT method. HepG2 cells, differentiated 3T3-L1 cells, and differentiated L6 cells were incubated with H-1-2 at different doses for 12, 24, and 48 h. Cellular viability was negatively correlated with the dose of H-1-2. Compared to the control group, cellular viability obviously decreased by over 11.78% (*p* < 0.01) when the dose of H-1-2 was no less than 600 μg/mL; however, there were no changes (*p* > 0.05) at concentrations between 50 and 600 μg/mL. The results of the MTT test are given in Figure 6.

Rosiglitazone is a member of the thiazolidinedione class of drugs, which act as insulin sensitizers. They reduce glucose, fatty acid, and insulin blood concentrations. They work by binding to the peroxisome proliferator-activated receptors (PPARs). PPARs are expressed in fat cells, cells of the liver, muscle, heart, and inner wall (endothelium), and the smooth muscle of blood vessels. There are several PPARs, including PPARα, PPARβ/δ, and PPARγ. Thiazolidinediones bind to PPARγ. PPARγ is expressed mainly in fat tissue, where it regulates genes involved in fat cell (adipocyte) differentiation, fatty acid uptake and storage, and glucose uptake. Rosiglitazone is markedly effective in reducing liver fat content by 30%–50% and sensitizes the liver to insulin. This reduces the amount of endogenous and exogenous insulin needed to inhibit hepatic glucose production [20]. Thus, we performed a cellular glucose consumption test using rosiglitazone as a positive control to confirm the effects of H-1-2.

**Figure 7:**
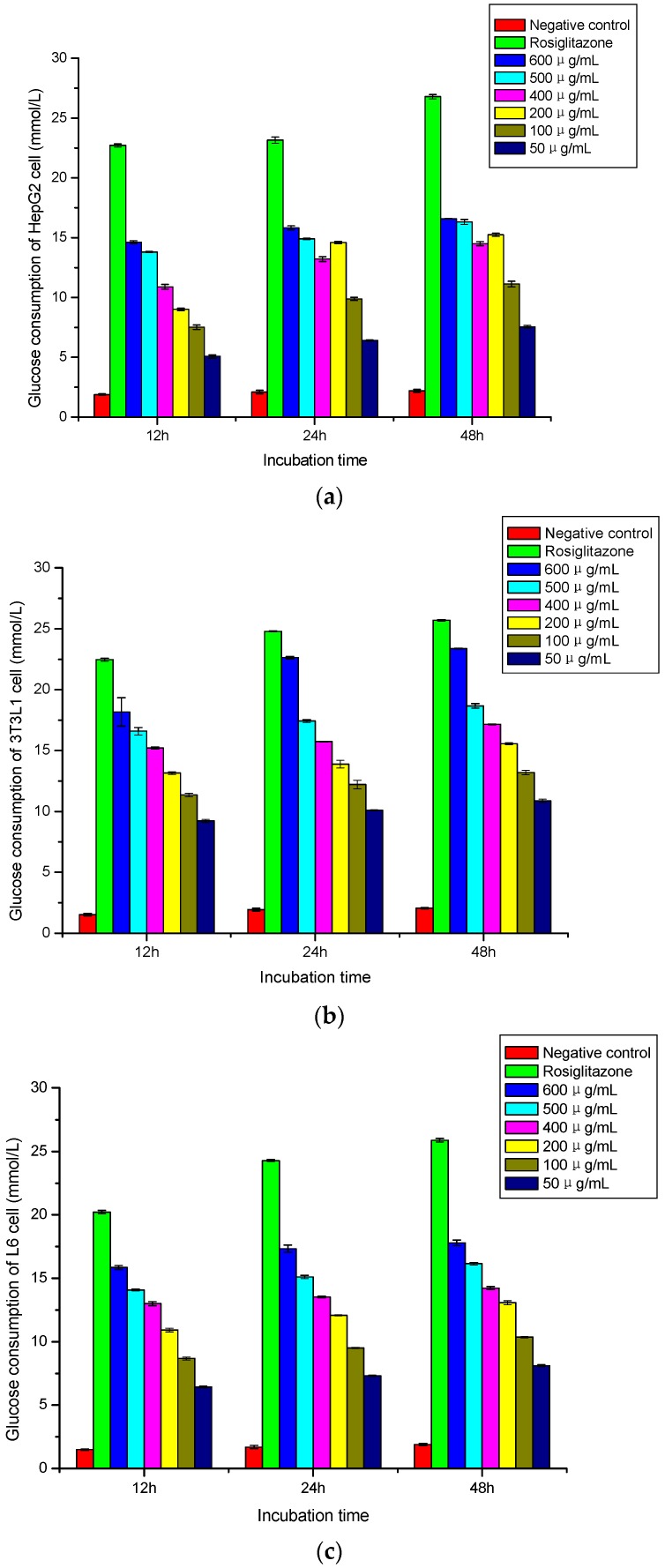
(**a**) Influence of H-1-2 on HepG2 cell glucose consumption at different incubation times (mean ± standard deviation, *n* = 5); (**b**) influence of H-1-2 on 3T3L1 cell glucose consumption at different incubation times (mean ± standard deviation, *n* = 5); and (**c**) influence of H-1-2 on L6 cell glucose consumption at different incubation times (mean ± standard deviation, *n* = 5).

Three cell lines were incubated with H-1-2 at different concentrations (50 μg/mL, 100 μg/mL, 200 μg/mL, 400 μg/mL, 500 μg/mL, and 600 μg/mL) and for different amounts of time (12 h, 24 h, and 48 h). H-1-2 was able to increase the glucose consumption of HepG2, 3T3 L1, and L6 cells (*p* < 0.01) at 12 h, while at both 24 h and 48 h, and after 48 h, the cultured cells showed a declining tendency to proliferate. HepG2, 3T3 L1, and L6 cells were incubated for 24 h in a blank control, and the glucose consumption was 2.10 ± 0.16, 1.93 ± 0.11, and 1.69 ± 0.13 mmol/L, respectively. Rosiglitazone can significantly increase the glucose consumption of HepG2, 3T3 L1, and L6 cells (*p* < 0.01): after incubation 24 h with rosiglitazone at a concentration of 100 μM, glucose consumption was 23.16 ± 0.27, 24.78 ± 0.04, and 24.28 ± 0.08 mmol/L, respectively. Incubation with H-1-2 at the concentrations of 50–600 μg/mL for 24 h showed that glucose consumption of HepG2, 3T3 L1, and L6 cells grew from 6.41 ± 0.05 to 15.83 ± 0.16 mmol/L, 10.09 ± 0.05 to 22.62 ± 0.09 mmol/L, and 7.32 ± 0.05 to 17.33 ± 0.29 mmol/L, respectively, in a dose-dependent manner. More extensive tabulations are illustrated in Figure 7a–c.

#### 2.6.2. Effects of H-1-2 on Glucose Uptake

**Figure 8:**
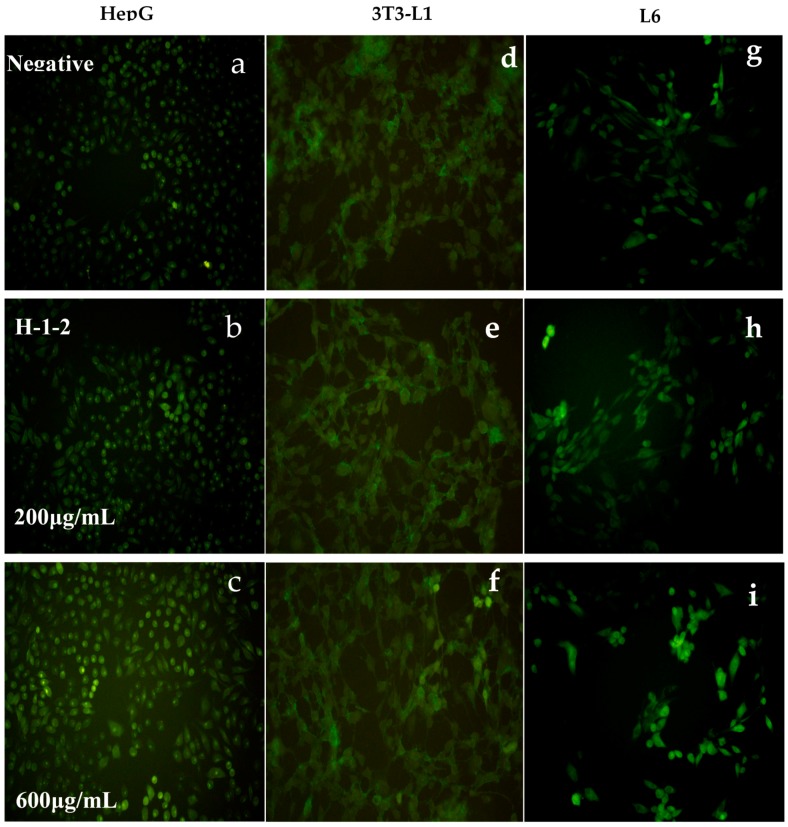
Fluorescence micrographs of 2-NBDG uptake into HepG2, 3T3-L1, and L6 cells incubated using H-1-2 for 48 h: (**a**) 2-NBDG uptake into HepG2 cells (blank control); (**b**) 200 μg/mL H-1-2 influence on 2-NBDG uptake into HepG2 cells; (**c**) 600 μg/mL H-1-2 influence on 2-NBDG uptake into HepG2 cells; (**d**) 2-NBDG uptake into 3T3L1 cells (blank control); (**e**) 200 μg/mL H-1-2 influence on 2-NBDG uptake into 3T3L1 cells; (**f**) 600 μg/mL H-1-2 influence on 2-NBDG uptake into 3T3L1 cells; (**g**) 2-NBDG uptake into L6 cell (blank control); (**h**) 200 μg/mL H-1-2 influence on 2-NBDG uptake into L6 cells; and (**i**) 600 μg/mL H-1-2 influence on 2-NBDG uptake into L6 cells; (*n* = 5).

Three cell lines were incubated with H-1-2 at different concentrations (50 μg/mL, 100 μg/mL, 200 μg/mL, 400 μg/mL, 500 μg/mL, and 600 μg/mL) and at different times (12 h, 24 h, and 48 h), and fluorescent d-glucose analog 2-[*N*-(7-nitrobenz-2-oxa-1,3-diazol-4-yl)amino]-2-deoxy-d-glucose (2-NBDG) was added as a fluorescent indicator for incubated cell for 60 min. H-1-2 increased glucose uptake in HepG2, 3T3-L1, and L6 cells. The escalating rate of glucose uptake became greater as the concentrations increased. H-1-2 was able to significantly improve cellular glucose consumption (*p* < 0.01) at 12 h; at both 24 h and 48 h, H-1-2 clearly increased cellular glucose uptake (*p* < 0.05); and, after 48 h, the cultured cells showed a declining tendency to proliferate. At 48 h, H-1-2, at all doses, prominently enhanced HepG2, 3T3-L1, L6 cellular glucose uptake rates from 10.37% to 30.02%, 6.17% to 19.21%, and 7.73% to 19.92%, respectively. Detailed experimentation results are listed in Table 3, and only partial fluorescence micrographs are shown in Figure 8.

### 2.7. Distribution of H-1-2 Taken Orally in Abdominal Organs in Rat

**Figure 9:**
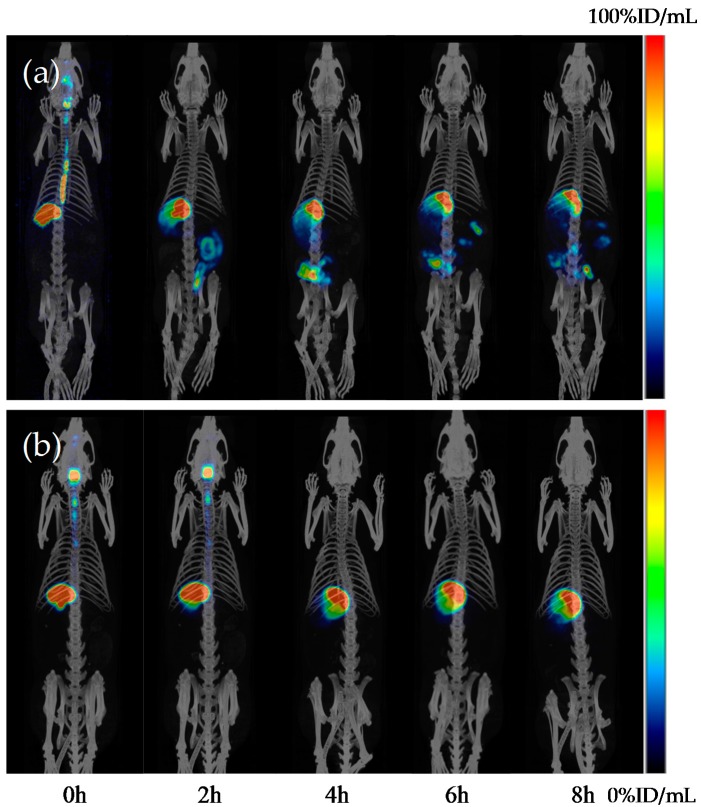
3D-SPECT scaning images: (**a**) Different organ uptake values of ^99m^Tc-labeled H-1-2, taken orally, in rats, propagated from the esophagus, stomach, small intestine, the large intestine, and then to the bladder with time. The gastric emptying time for H-1-2 was about 8 h; (**b**) Contrast group free technetium propagated from the esophagus, a major amount of the radioactivity was concentrated in the stomach and there was no uptake into the intestine; *n* = 3.

^99m^Tc was used to label homogeneous polysaccharide H-1-2. The results obtained from TLC indicated that the labeling efficiency of ^99m^Tc to H-1-2 was approximately 98%. The ^99m^Tc-labeled H-1-2 was used for SPECT/CT studies to investigate the distribution of H-1-2, taken orally, in rat. As shown in Figure 9, the radioactivity (the drug carrier, H-1-2) propagated from the esophagus, stomach, small intestine, the large intestine, and then to the bladder with time. The gastric emptying time for the H-1-2 was about 8 h; at that time, the radioactivity observed in the small intestine increased significantly. At 4 h post ingestion, about 50% of the radioactivity was observed in the intestine. No significant radioactivity was found in the heart, liver, and kidney, conjecturing absorption of ^99m^Tc-labeled H-1-2 might travel through intestinal mucosa absorption into systemic circulation. This problem will need further investigation. However, free technetium contrast group propagated from the esophagus, a major amount of the radioactivity was concentrated in the stomach and there was no uptake into the intestine. The distribution of H-1-2 taken orally in different abdominal organs, such as stomach, intestine, heart, liver, kidney, and bladder, in rats, are shown in Table 4 and Table 5.

## 3. Experimental Section

### 3.1. Plant Materials

*Pseudostellaria heterophylla* (Miq.) Pax medicinal slices were purchased from Fujian Xiang-An drug limited company (Quanzhou, Fujian, China). They were cut into pieces, oven dried below 60 °C, and then crushed into powder.

### 3.2. Chemicals

HPLC grade methanol, acetonitrile, was bought from Merck (Darmstadt, Germany). Analytical grade reagents were bought from Sinopharm Chemical Reagent Co., Ltd. (Shanghai, China). Sugar standards and DMSO were purchased from Sigma-Aldrich (Shanghai, China). Dextran T-2000, T-700, T-500, T-200, T-80, T-70, T-40, and T-10 were produced by Pharmacia (Stockholm, Sweden). Whatman DEAE-cellulose 52 and Sephacryl S-300 was purchased from GE Healthcare Technology, (Uppsala, Sweden). *N*-cyclohexyl-*N*′-[2-(*N*-methyl-morpholino) ethy] carbodiimide-4 (CMC) was purchased from Tokyo Chemical Industry (Tokyo, Japan). Penicillin/streptomycin solution, 0.25% Tripsin-EDTA, and PBS were purchased from Nanjing Keygen Biotech. Co., Ltd. (Nanjing, China). GIBCO DMEM was produced by Invitrogen (Grand Island, NY, USA). Water was deionized using the Milli-Q-Plus ultra-pure water system (Milford, MA, USA).

### 3.3. Cells

HepG2 cells (human liver hepatocellular) and 3T3-L1 cells (mouse pre-adipose embryo fibroblast) were purchased from Nanjing Keygen Biotech. Co., Ltd. (Nanjing, China). L6 cells (rat skeletal muscle cell line) were purchased from Shanghai Bioleaf Biotech Co., Ltd. (Shanghai, China).

### 3.4. Isolation and Purification of Pseudostellaria heterophylla Polysaccharides

Raw polysaccharide was extracted from *Pseudostellaria heterophylla* using hot water extraction methods [11]. After this, it was treated with deproteinization, water dialysis, DEAE-cellulose 52, and Sephacryl S-300 column chromatography grading. The results showed that papain combined with Sevag presented the optimum deproteinizing conditions. The *Pseudostellaria heterophylla* polysaccharides were eluted with water, 0.05, 0.1, 0.2, and 0.5 mol/L NaCl salt solution using DEAE-Cellulose 52 column grading; finally, the main components were eluted with water by Sephacryl S-300 grading.

### 3.5. Analytical Methods

#### 3.5.1. Polysaccharide Content, Monosaccharide Composition, and Molecular Weight Distribution Determination

Polysaccharide content was determined using the phenol-sulfuric acid method with d-glucose as a standard. Determination of monosaccharide composition was performed according to previous methods [21]. Polysaccharide molecular weight was assayed by the HPGPC method using an Agilent Technologies 1260 series (Agilent Co., Santa Clara, CA, USA) apparatus with RID detectors equipped with two columns in series, and procedures were performed according to previous methods [22]. Both Ultrahydrogel^TM^ 2000 and Ultrahydrogel^TM^ 250 column sizes were 7.8 mm × 300 mm (Waters Technologies, Milford, MA, USA). The H-1-2 was dissolved in distilled water at 5 mg/mL, with 0.1 mol/L sodium nitrate as the mobile phase, with a flow rate of 0.6 mL/min. The column temperature was maintained at 30 °C, and the refractive index detector was used (detector temperature, 35 °C) to inject a sample volume of 20 μL; prior to injection into the HPGPC system, it was filtered through a 0.45-μm membrane. Dextran MW standards, ranging from 1.0 to 200 kDa (Pharmacia, Stockholm, Sweden), were used to plot the lgM-t (mean molecular weight-retention time) calibration curve.

#### 3.5.2. Sequence of Polysaccharide Determination

##### Periodate Oxidation

To 50 mg of H-1-2 in distilled water, 30 mmol/L of sodium metaperiodate (30 mmol/L NaIO_4_) was added to reach a total volume of 50 mL. The solution was kept in the dark, at room temperature, and the consumption of periodate was followed using a spectrophotometer. Drawing 0.1 mL from each tube at 72 h, and diluted, respectively, to 25 mL, was used to determine the value of the optical density at 223 nm. The excess periodate was destroyed by the addition of glycol to terminate the reaction when the value of the optical density at 223 nm was invariable. The amount of periodate consumed in the reaction can be seen in the standard curve by the absorbency measured. Two milliliters of the reaction solution were drawn and one, 2-mL drop of bromocresol blue was added to the reaction solution, and then titrated with 0.005 mol/L NaOH to determine the amount of formate produced in the reaction. The remnant was used for Smith hydrolysis [23].

##### Smith Hydrolysis

The products of periodate oxidation were dialyzed against flowing water for 48 h, and distilled water for 24 h. Concentration was performed under reduced pressure at bath temperatures below 40 °C, to about 10 mL of the total volume. It was reduced with 70 mg of potassium borohydride (KBH_4_), stirred in the dark, at room temperature, for 18–24 h. The solution was neutralized with 0.1 mol/L acetic acid to pH 6–7, dialyzed against flowing water for 48 h, and distilled water for 24 h, and concentrated under reduced pressure until dry. Two milliliters of 1 mol/L H_2_SO_4_ was added to the ampoule and it was sealed, hydrolyzed at 100 °C for 8 h. The hydrolysate was neutralized with BaCO_3_, the filtrate was filtered and collected, and then concentrated to be determined using paper chromatography [24].

##### Methylation Analysis

Vacuum-dried H-1-2 (8 mg) was solublized in 2 mL of dimethyl sulfoxide (DMSO) in the ampoule, and then it was sealed and stirred with a wire whisk until it was completely dissolved. Twenty milligrams of solid NaOH were added and stirred for 15 min. The derivatization was triggered by loading 0.3 mL of cold CH_3_I, dropwise, until it was fully cooled; then, it was stirred for at least 30 min. Superfluous CH_3_I was removed at low pressure by a rotary evaporator. After dialysis and lyophilization, the methylated polysaccharides were hydrolyzed with 3 mL of 2 mol/L trifluoacetic acid. A total of 20 mg of NaBH_4_ was added to reduce the hemiacetal group. After incubation at 25 °C for 2 h, 100 μL of glacial acetic acid was used to terminate the reduction. A Finnigan MD-800 GC-MS system (San Francisco, CA, USA) was used to analyze the glycosidic linkage. The acetylated derivatives were loaded into a HP-1 capillary column (25 m × 0.25 mm, I.D). The temperature program was set as follows: The initial temperature of the column was 180 °C at 13 min and increased to 260 °C at 20 min; injection temperature was 230 °C. The ion source of the mass spectrometer was set at 260 °C [25].

#### 3.5.3. Polysaccharide Structure Analysis

##### Fourier Transforms Infrared Analysis

Up to 2 mg of dried *Pseudostellaria heterophylla* polysaccharide, H-1-2, was added to 200 mg of KBr powder, gently ground in an agate mortar, and then pressed into KBr tablets. Subsequently, the tablets were scanned from 4000 to 400 cm^−1^ using a Thermo Scientific Nicolet iS5 Fourier Transform Infrared Spectrometer (Madison, WI, USA). The methylated polysaccharide samples were determined, using paraffin oil to make the film (the Nujol method) [26].

##### Nuclear Magnetism Spectra Analysis

Structure analyses were performed using NMR analyses with a Bruker Avance III spectrometer at 500 MHz, equipped with a ^13^C/^1^H dual probe in FT mode (Bruker, Stockholm, Sweden). H-1-2 was previously dissolved in deuterium (D_2_O, 99.9%) and lyophilized three times to replace exchangeable protons with deuterium. The lyophilized samples were then dissolved in D_2_O at a concentration of 40–60 mg/mL. All spectra were recorded using HOD suppression by presaturation. The interpretations of the ^1^H/^1^H correlated spectroscopy (COSY), ^1^H/^13^C heteronuclear single-quantum coherence (HSQC), and heteronuclear multiple bond coherence (HMBC) spectra were recorded using a state-time proportion phase incrementation for quadrature detection in the indirect dimension. ^13^C spectrum was determined with acetone as the internal standard (methyl C atomic chemical shift of 31.5 ppm), and the ^1^H spectrum was done with the HOD peak as calibration standard (δ 4.85 ppm) [27,28].

### 3.6. Determination of Glucose Consumption and Glucose Uptake in Cells

Effect of H-1-2 on the activity of hepG2 cells, 3T3-L1 cells, and L6 cells were determined using the MTT method assay. Glucose consumption and glucose uptake in hepG2 cells, 3T3-L1 cells, and L6 cells were measured using the glucose oxidase method [29], and the transport of glucose used a fluorescence probe (2-NBDG), respectively [30].

### 3.7. Animal Study—Biodistribution of H-1-2

Six Sprague Dawley (SD) male rats, weighing between 150–180 g, were used. Food and water were available ad libitum. This study was approved by the Fujian University of Traditional Chinese Medicine Ethics Committee. The biodistribution of the H-1-2 carrier in rats was studied using single-photon emission computed tomography (SPECT)/computed tomography (CT) after oral administration of a test polysaccharide. H-1-2 was labeled with sodium pertechnetate (^99m^Tc) using a stannous chloride (SnCl_2_) method [31]. DMSO solution H-1-2 (10 mg/mL pH 6.0) was mixed with 300 mL of NaCl aqueous ^99m^Tc (20–30 mCi) in the presence of 20 mL of HCl aqueous SnCl_2_ (2 mg/mL) reaction, at 50 °C for 60 min. The labeling efficiency of ^99^mTc to CS was assessed using thin layer chromatography (TLC), using silica gel coated TLC plates (Ann Arbor, MI, USA). TLC was performed using aqueous NaCl as the mobile phase.

A three-dimensional registration method for automated fusion of MILabs Micro PET-CT-SPECT whole-body images (Heidelberglaan, The Netherlands) was used for animal imaging. This system features helical scanning for both SPECT and CT acquisitions, using a translation stage with variable axial ranged scanning. Animal imaging was performed under a controlled temperature (37 °C) and anesthesia was used (1.5% isoflurane). The dynamic scans of rats were acquired immediately after oral administration of the ^99m^Tc-labeled H-1-2 (100 MBq, 1 mL) and an equal amount of MBq free technetium was used as blank control. Acquisition time points were 0 h, 2 h, 4 h, 6 h, and 8 h, and the collection method for static 15 min SPECT, in the resolution of the whole body CT and analyzed using software by PMOD Technologies Ltd., (Zürich, Switzerland), version 3.6, for quantitative analyses of uptake into the various organs of the animals.

### 3.8. Statistical Analyses

All data were treated using SPSS15.0 for statistical analyses. Basic data were analyzed by means of *t*-test or ANOVA, and one-way ANOVA was used for comparing more than two independent means. All values are expressed as mean ± SD, while *p* < 0.05 indicated a significant difference.

## 4. Conclusions

In this paper, a novel homogeneous polysaccharide, named H-1-2, was isolated from *Pseudostellaria heterophylla* (Miq.) Pax ex Pax et Hoffm. The mean molecular weight of H-1-2 was 1.4 × 10^4^ Da. H-1-2 contained only d-glucose. H-1-2 had a specific rotation of [α]${}_{D}^{25}$ + 153.0° (*c* = 0.510, H_2_O). The chemical structure of the H-1-2 polysaccharide resembles that of amylase starch. The structure of H-1-2 contains pyranride, and has the characteristics of an α-iso-head configuration. In addition, it may be a neutral polysaccharide. H-1-2 contains a non-reducing end (T-), 1,4-, 1,6-, and 1,4,6-connections; four ways to connect glucose. H-1-2 is a type of glucan where the chemical combination exists in the main chain, between 1→4 linked glucose, and contains a small amount of 1,6-linked glucose in the branched chain.

HepG2, 3t3-L1, and L6 cell lines were used to assess cellular glucose consumption and cellular glucose uptake in vitro. H-1-2 could clearly increase cellular glucose consumption and cellular glucose uptake in a dose-dependent manner. H-1-2 can increase glucose uptake and utilization in muscle and adipose cells, and it is beneficial to screen for in the discovery of anti-diabetes lead compounds. H-1-2 was effectively similar to a conventional, anti-diabetic agent, rosiglitazone. To better understand how H-1-2 can best be used in the development of new medicines, which can be exploited in the search for new treatments for diabetes, focusing on the glucose uptake in skeletal muscle or adipose depots is recommended [32].

The oral absorption mechanisms of polysaccharide are unknown. H-1-2 was labeled with radioisotopes (^99m^Tc-pertechnetate). ^99m^Tc-labeled-H-1-2 was checked using SPECT/CT analysis images, after oral administration, in rats. At 4 h post ingestion, about 50% of the radioactivity was observed in the intestine. No significant radioactivity was found in the heart, liver, and kidney, conjecturing that absorption of ^99m^Tc-labeled H-1-2 might be via the intestinal mucosa into systemic circulation. The problem of whether polysaccharides are absorbed after oral ingestion, via the intestinal paracellular pathway, needs further research.

## Figures and Tables

**Table 1:** The methylation reaction results of H-1-2.

Methylated Glycosyl	Retention Time	Mole Ratio	Mass Charge Ratio (*m*/*z*)	Link Mode
2,3,4,6-Me_4_-Glc	14.275	1	43, 71, 87, 101, 117, 129, 145, 161, 205	Glc(1→
2,3,6-Me_3_-Glc	18.522	14	43, 71, 87, 99, 101, 117, 129, 161, 233	→4)Glc(1→
2,3,4-Me_3_-Glc	19.075	1.5	43, 71, 87, 101, 117, 129, 161, 189, 233	→6)Glc(1→
2,3-Me_2_-Glc	22.958	1.2	43, 85, 99, 101, 117, 127, 159, 201, 261	→4,6)Glc(1→

**Table 2:** ^1^H-NMR and ^13^C-NMR chemical shift correlation values of H-1-2.

Glycosy Link Mode	Chemical Shift (ppm)					
	H-1/C-1	H-2/C-2	H-3/C-3	H-4/C-4	H-5/C-5	H-6/C-6
Glc(1→	5.44/100.83	3.66/72.97	3.98/73.95	3.45/70.55	4.02/72.41	3.88/61.65
→4)Glc(1→	5.44/100.83	3.70/72.77	4.0/74.60	3.68/77.91	3.86/72.41	3.88/61.65
→6)Glc(1→	5.01/98.93	3.63/72.64	3.75/74.35	3.54/70.76	3.93/71.49	3.78/66.73
→4,6)Glc(1→	5.01/98.93	3.55/72.64	3.70/74.13	n.d./77.91	n.d./71.49	3.78/66.73

Note that for ^13^C-NMR, acetone was used as internal standard, δ 31.5 ppm; and for ^1^H-NMR HOD, was used as standard, δ 4.85 ppm.

**Table 3:** Effects of H-1-2 on cellular glucose uptake (*n* = 5).

Cells		HepG2			3T3L1			L6		
Group (μg/mL)		Escalating Rate (%)								
		12 h	24 h	48 h	12 h	24 h	48 h	12 h	24 h	48 h
H-1-2	50	8.06	9.92	10.37	2.52	4.68	6.17	3.78	3.92	7.73
	100	12.20	14.06	16.93	5.21	5.90	9.11	4.81	5.30	7.84
	200	15.77	17.03	22.88	5.80	9.32	12.10	5.94	7.23	10.91
	400	18.09	20.39	23.96	8.78	10.22	15.16	8.41	8.66	12.29
	500	19.89	21.74	26.11	9.75	11.91	16.44	9.16	10.80	19.07
	600	23.21	23.95	30.02	10.91	13.08	19.21	10.29	14.69	19.92

**Table 4:** Different organ uptake values of ^99m^Tc-labeled H-1-2, taken orally, in rats (*n* = 3).

Times	Uptake Values of ^99m^Tc-Labeled H-1-2 (ID%/mL)					
	Stomach	Intestine	Heart	Liver	Kidney	Bladder
0 h	50.725	0.000	0.002	0.025	0.026	0.012
2 h	19.057	13.123	0.050	0.026	0.029	0.024
4 h	18.750	16.456	0.060	0.018	0.054	0.008
6 h	18.191	6.264	0.026	0.022	0.114	0.015
8 h	18.074	3.664	0.048	0.016	0.112	0.014

**Table 5:** Different organ uptake values of free technetium, taken orally, in rats (*n* = 3).

Times	Uptake Values of Free Technetium (ID%/mL)					
	Stomach	Intestine	Heart	Liver	Kidney	Bladder
0 h	36.527	0.000	0.028	0.042	0.100	0.028
2 h	34.240	0.025	0.039	0.041	0.132	0.044
4 h	24.566	0.057	0.054	0.084	0.090	0.157
6 h	22.249	0.131	0.010	0.057	0.097	0.108
8 h	20.229	0.033	0.008	0.147	0.064	0.066
